# Domestication Syndrome in *Dacryodes edulis* (Burseraceae): Comparison of Morphological and Biochemical Traits between Wild and Cultivated Populations

**DOI:** 10.3390/plants11192496

**Published:** 2022-09-23

**Authors:** Franca Marcelle Meguem Mboujda, Marie-Louise Avana-Tientcheu, Stéphane Takoudjou Momo, Alix Mboukap Ntongme, Virginie Vaissayre, Laura N. Azandi, Stéphane Dussert, Hilaire Womeni, Jean-Michel Onana, Bonaventure Sonké, Christopher Tankou, Jérôme Duminil

**Affiliations:** 1 Research Unit of Fauna, Sylviculture of Wood Technology, Faculty of Agronomy and Agricultural Sciences, University of Dschang, Dschang BP: 222, Cameroon; 2 DIADE, IRD, University of Montpellier, CIRAD, 34394 Montpellier, France; 3 Plant Systematic and Ecology Laboratory, Higher Teacher’s Training College, University of Yaoundé I, Yaoundé P.O. Box 047, Cameroon; 4 AMAP, IRD, CNRS, INRAE, CIRAD University of Montpellier, 34398 Montpellier, France; 5 Research Unit of Biochemistry, Medicinal Plants, Food Science and Nutrition, Faculty of Science, University of Dschang, Dschang BP: 67, Cameroon; 6 Evolutionary Biology and Ecology, Faculté des Sciences, C.P. 160/12, Université Libre de Bruxelles, 50 Avenue F. Roosevelt, 1050 Brussels, Belgium; 7 Department of Plant Biology and Physiology, Faculty of Science, University of Yaoundé I, Yaoundé P.O. Box 1601, Cameroon; 8 Department of Crop Sciences, Faculty of Agronomy and Agricultural Sciences, University of Dschang, Yaoundé BP: 222, Cameroon

**Keywords:** African plum tree, domestication syndrome, evolutionary history, fruit tree species, non-timber forest products

## Abstract

For millennia, people have harvested fruits from the wild for their alimentation. Gradually, they have started selecting wild individuals presenting traits of interest, protecting and cultivating them. This was the starting point of their domestication. The passage from a wild to a cultivated status is accompanied by a modification of a number of morphological and genetic traits, commonly known as the domestication syndrome. We studied the domestication syndrome in *Dacryodes edulis* (G.Don) H.J.Lam (known as ‘African plum’ or ‘safoutier/prunier’), a socio-economically important indigenous fruit tree species in West and Central Africa. We compared wild and cultivated individuals for their sex distribution; flower, fruit and seed morphometric characteristics; seed germination temporal dynamic and fruit lipid composition. We found a higher percentage of male and male-hermaphrodite sexual types in wild populations than in cultivated ones; a lower fruit and seed mass in wild individuals; and similar mean time of germination, oil content and fatty acid composition between wild and cultivated individuals. Our results are interpreted in light of the presence of a domestication syndrome in *D. edulis*.

## 1. Introduction 

Crop domestication is a long-term evolutionary transformation of wild individuals into cultivated ones by a continuous selection of desired traits of the species in order to satisfy people’s preferences and needs [1]. The domestication process can be seen as a continuous process that starts with species maintenance and care in their natural environment [2]. It is then followed by species cultivation in human environments using material from wild populations [3], involving a generation-to-generation selection of traits of interest to humans [2,4]. Clement defined five stages during the domestication process where the differentiation between wild and domesticated populations occurred along a continuum from minor to extreme, respectively, for incipient to full domesticates [4]. 

The development of agricultural practices in the Central African rainforests probably began in the late Stone Age (ca. 7000 BP) and increased in intensity and scope during the Neolithic (ca. 4000–2500 BP) and the Iron Age (2500–500 BP) stages with the development of new agricultural practices involving notably the exploitation of arboreal resources [5,6]. This is notably supported by the presence of charred archaeobotanical remains of culturally valuable species in ancient human settlements (e.g., soil charcoals and endocarps of *Elaeis guineensis* and *Canarium schweinfurthii*) [5,6,7,8]. Species’ domestication’s evolutionary history can be studied by comparing morphological and genetic traits between cultivated and ancestral wild populations. Observed differences most probably result from human selection on cultivated material and to new environmental conditions encountered by cultivated populations. Both human and environmental factors generate selective pressures that affect the phenotypes of cultivated individuals, leading to morphological and genetic divergences from their wild ancestors [9,10]. Moreover, genetic variability in cultivated populations is generally reduced in comparison to wild populations due to the selection of a limited number of wild individuals with interesting traits for their introduction into cultivation, process better known as the domestication bottleneck [11]. On the other hand, cultivation generally results in an improvement of users’ preferred traits (e.g., fruit and seed size) in cultivated populations as compared to their wild ancestral populations [12]. Thus, fruits from cultivated trees are often larger, more variable in shape and color, and taste better (e.g., sweeter, less acid, astringent, etc.) than those from their wild ancestors [3,13]. The taste is associated with the biochemical characteristics of the fruits, thus, wild and cultivated fruits generally diverge for these characteristics [14,15]. Moreover, for the specific case of dioecious species, domestication can imply a modification of the breeding system with an evolution toward gynodioecy, andromonoecy or hermaphroditism [9,10]. 

*Dacryodes edulis* (G.Don) H.J.Lam (Burseraceae)—known as safoutier, prunier or atangatier in French, or as African plum or African pear tree in English—is an emblematic food tree species indigenous to Central Africa, from Nigeria in the West to Angola in the South and Democratic Republic of Congo in the East. This multipurpose tree provides food (fruits eaten raw or cooked), medicine (leaves, resins and bark) or cash income to rural populations (trade of fruits) [16,17]. The species has probably been used for centuries/millennia [18]. When cultivated, *D. edulis* starts flowering 4–5 years after planting (Kengue 1990). It most certainly originated from dense evergreen and semi-deciduous rain forests at low altitudes and was introduced in agrosystems by locals many generations ago. It grows at an optimal temperature of 23–25 °C, a rainfall between 1400 and 2500 mm per year and an altitude of up to 1000 m [19]. The species can reach more than 20 m in height in its natural environment and up to 100 cm in trunk diameter [20]. *Dacryodes* species are known as dioecious species, yet cultivated *D. edulis* individuals either present male and hermaphrodite flowers in varying proportion or present only female flowers [21]. Previous studies on its floral biology indicated that male, female or hermaphrodite flowers are in no particular arrangement and in varying proportions from one inflorescence (measuring 15–40 cm) to the other, from one year to the other and from one tree to the other [21]. Some research works have been conducted on the reproductive system of *D. edulis* [22,23], the characterization of its fruits and seeds as well as their oil content/fatty acids composition [24,25,26,27], but focusing only on cultivated populations of the species. 

For several decades, the domestication of *D. edulis* has been promoted as a strategy to diversify production systems and improve the quality and quantity of its production in farmers’ fields [28]. Such a strategy is visible in the field, with an observed improvement in some preferred traits such as fruits’ size in some producing areas, due to farmers-led selection across generations of trees with valued traits for consumption and markets [24,28]. The presence of this phenotypic variation in the field support the hypothesis of a domestication syndrome oriented by farmers’ management practices and expressed through the dominance of certain traits favored during the process of intensification of the species’ cultivation [29]. In order to test this hypothesis, we conducted an integrative study comparing the sex distribution, flower, fruit and seed morphometric characteristics, seed germination temporal dynamic, oil content and fatty acid composition between wild and cultivated *D. edulis* individuals. These traits are selected during the process of domestication of fruit trees [9].

## 2. Results

### 2.1. Sex Distribution in D. edulis 

We found four sexual types (male, female, male-hermaphrodite and hermaphrodite) in cultivated individuals, and three (male, female and male-hermaphrodite) in the wild ones (Table 1; Figure S1; see Supplemental Materials with this article). Some cultivated individuals were bearing only hermaphrodite flowers (no male flowers found), which might also have resulted from a methodological bias (only two inflorescences characterized per tree with little chance to sample male flowers). The male function was thus accomplished by trees bearing male flowers only, both male and hermaphrodite flowers and hermaphrodite flowers only, whereas the female function was accomplished by trees bearing female flowers only, male-hermaphrodite and hermaphrodite flowers. The distribution of the male/female function were 65.2%/82.6% in cultivated individuals and 79%/52.6% in wild individuals.

**Figure 1:**
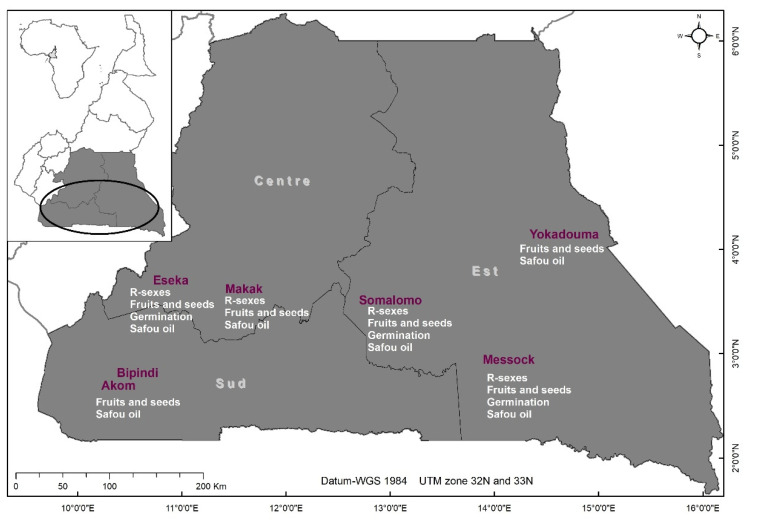
Study sites in the Centre, South and East regions of Cameroon and list of traits studied in each site to assess the domestication syndrome in *D. edulis* (R-sexes: sex distribution; fruits and seeds: fruit and seed morphometrics; germination: seed germination tests; safou oil: mesocarp lipid content and fatty acid composition).

The MFA (Mixed Factor Analysis) explained 42% of the total variance with 26.31% for axis 1 and 12.53% for axis 2 (Figure S2A). Axis 1 discriminated the wild and cultivated individuals (Figure S2C), with wild individuals (Somalomo and Messock; Figure 1) being mostly male or male-hermaphrodite, whereas cultivated individuals (Makak and Eseka) were mostly female or hermaphrodite. This discrimination along axis 1 was associated with variables related to ovaries and flowers (Figure S2B). On the other hand, axis 2 allowed us to discriminate individuals according to dendrometric parameters (tree height and dbh) associated with variables related to the stamina and inflorescence (Figure S2D).

### 2.2. Morphometric Characteristics of Flowers according to the Sexual Type, Status of the Individuals and Sites 

Male flowers from wild trees had globally smaller stamens and higher peduncle length than cultivated ones (Table 2). However, this result was not clear-cut when considering the site level (values from Messock individuals were remarkably close to those from Eseka and Makak). The same pattern was observed for female flowers with the ovaries in wild flowers being globally smaller than those of cultivated ones (Table 2). At the site level, Messock presented intermediary values compared to Eseka and Makak. 

### 2.3. Morphometric Characteristics of Fruits and Seeds

**Figure 2:**
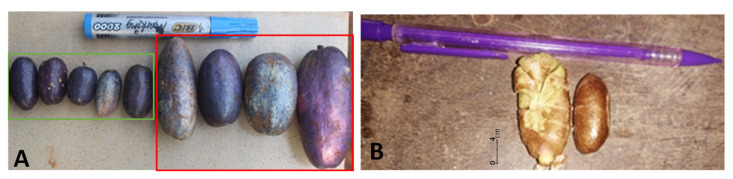
Morphological differences between wild and cultivated fruits and seeds of *D. edulis*. (**A**) Wild (squared in green) and cultivated fruits (squared in red); (**B**) a cultivated (left) and a wild seed (right).

We found significant differences in the morphology of wild and cultivated fruits (Table 3; Table S1). Globally, fruits from wild trees were characterized by a smaller mass, length, width and thickness than fruits collected from cultivated trees (Figure 2). Moreover, we observed that seeds from wild individuals had a smaller mass, length and width than those from cultivated trees. The distribution of the number of cotyledon lobes was similar for seeds from cultivated (6–17 cotyledon lobes) and wild trees (5–16) (Figure S3). We observed that 2% of the cultivated fruits were seedless.

**Figure 3:**
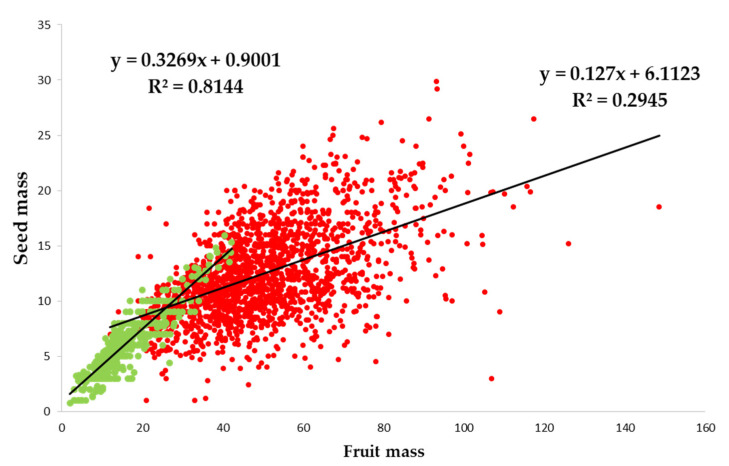
Correlation between fruit and seed mass for wild (in green) and cultivated (in red) individuals.

The first two axes of FAMD (Factor Analysis of Mixed Data) explained approximately 63.03% of the total variance of the morphological parameters of fruits and was divided into 45.68% for axis 1 and 17.35% for axis 2 (Figure S4). Axis 1 was able to discriminate individuals according to the status of individuals and sites (Figure S4A). This discrimination along axis 1 was achieved through variables related to fruit (fruit length, width and mass) and pulp (thickness and mass) characteristics (Figure S4B). Eseka and Makak were characterized by large fleshy fruits, Messock and Somalomo by small fleshy fruits with large seeds. Axis 2 discriminated individuals according to seed characteristics (number of cotyledon lobes, seed length and width) and fruit shape (Figure S4A). The wild trees were characterized by fruits that were spherical, whereas several fruit shapes (oval, oblong, conical) were observed for cultivated trees. Finally, we observed a stronger correlation between seed and fruit mass for wild individuals (R² = 0.81) than for cultivated ones (R² = 0.29) (Figure 3).

### 2.4. Seed Germination

**Figure 4:**
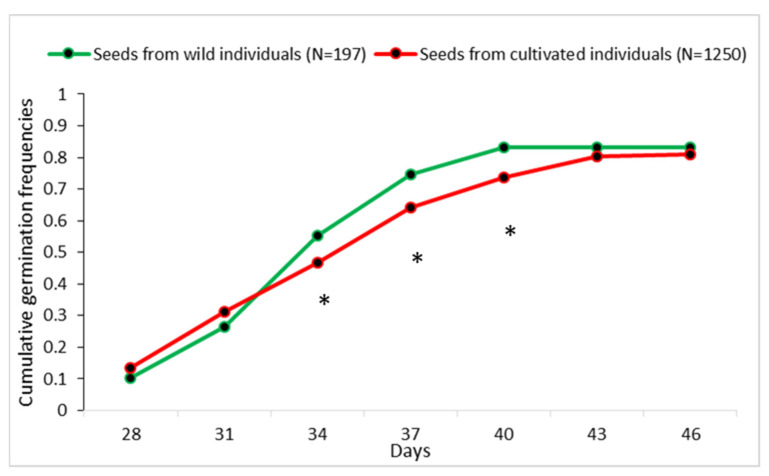
Cumulative germination frequencies of wild and cultivated seeds (* significantly different at *p* = 0.05; *t*-test).

Overall, the temporal dynamic was similar for seeds from wild and cultivated trees except between 34 and 40 days, where the germination rate was significantly higher for seeds from wild individuals than for those from cultivated ones. At 40 days after seed sowing, the germination rate (80% of the seeds) of wild seeds had reached its maximum whereas it was at 46 days for cultivated seeds (81%). The mean time of germination was of 34 ± 4.7days (mean ± standard deviation around mean) for seeds from wild trees and of 37 ± 6.5 days for those from cultivated ones (Figure 4), with no statistical difference (*p* = 0.4, *t*-test). 

**Figure 5:**
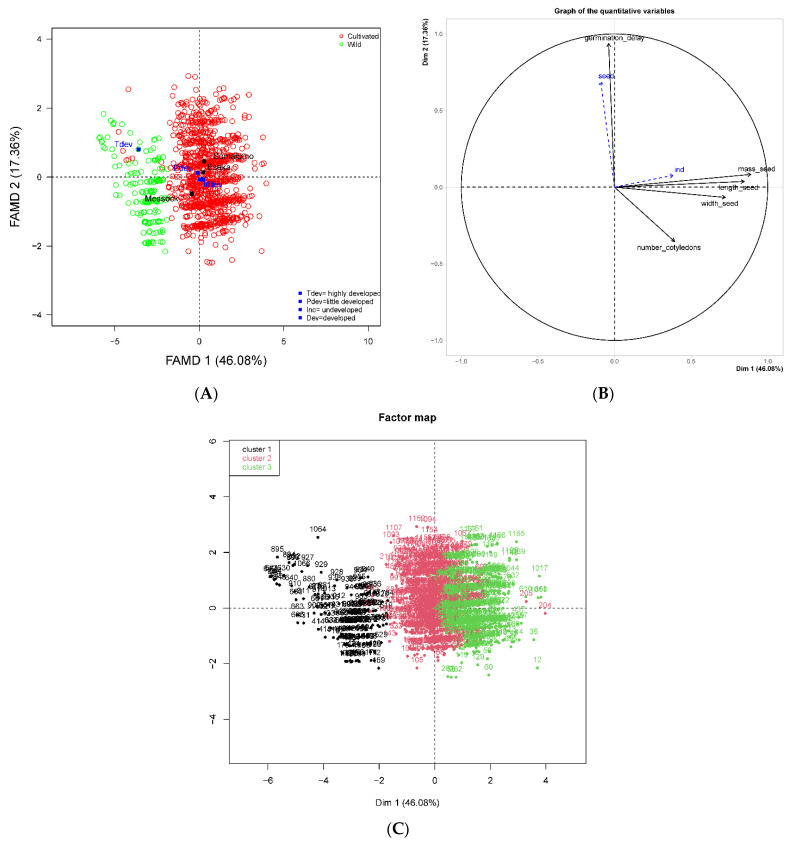
Classification of wild and cultivated seeds by FAMD analyses. (**A**) Discrimination of wild and cultivated seeds from the FAMD according to axes 1 and 2. (**B**) Correlation and contribution of variables to the formation of axes 1 and 2. (**C**) Clustering of seeds based on the wild and cultivated status of their mother’ trees.

The FAMD (first two axes) explained approximately 63.44% of the total variance and was divided into 46.08% for axis 1 and 17.36% in axis 2. Axis 1 could discriminate germination responses according to the status of individuals, sites and seed characteristics (Figure 5A). Axis 2 discriminated individuals according to germination time (Figure 5B). The classification (Figure 5C) of seeds showed three groups: cluster 1 with small seeds (from wild trees) with a short germination time and with low values for seed morphometric characteristics (length, seed width, seed mass and number of cotyledon lobes); cluster 2 had medium-size seeds with a germination time longer than seeds in cluster 1; and cluster 3 had high values for the morphometric parameters (seed length, seed width, seed mass and number of cotyledon lobes, and with the longest germination time). Clusters 2 and 3 were composed mostly of seeds from cultivated trees from Eseka and Somalomo.

### 2.5. Mesocarp Lipid Content and Fatty Acid Composition

**Figure 6:**
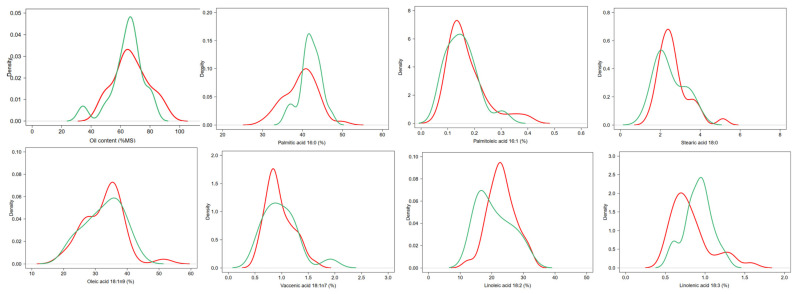
Distribution of the mesocarp oil content and fatty acid composition in cultivated (red lines) and wild (green lines) *D. edulis* individuals.

Total fruit mesocarp lipid content ranged from 34 to 81% in wild fruits and from 46 to 91% in cultivated fruits (Figure 6), with no significant difference between them (Table 4). Seven Fatty Acids (FAs) were identified and quantified (>0.1%) in all mesocarp oil samples: palmitic acid (16:0), palmitoleic acid (16:1), stearic acid (18:0), oleic acid (18:1n-9), vaccenic acid (18:1n-7), linoleic acid (18:2) and linolenic acid (18:3) (Figure 6). Palmitic, oleic and linoleic acids were the major FAs in all fruit oil samples studied (Table 4). For each major FA, a considerable variation was observed within both the cultivated and wild groups (Figure 6). For instance, oleic acid ranged from 21 to 42% in wild forms and from 19 to 51% in cultivated ones. However, the two groups did not significantly differ for the FA composition of mesocarp oil, except for palmitic acid, which showed a limited but significant difference between wild (42.0%) and cultivated (39.6%) trees (Table 4). PCA (Principal Component Analysis) separated individuals according to individual status, sites, mesocarp lipid content and fatty acid composition. The first two components explain ca. 66% of the total variance (46.56% for axis 1 and 19.24% for axis 2) (Figure S5). However, the PCA did not discriminate wild and cultivated individuals according to their mesocarp lipid content and fatty acid composition.

## 3. Discussion

### 3.1. Effect of Selection on Sex Distribution and Flower Morphometric Characteristics

The domestication syndrome involves a set of morphological and genetic modifications between the ancestral wild individuals of a species and its cultivated descendants. Sex distribution is one of the biological traits that can be modified. This is particularly relevant for dioecious species, where the presence of male individuals represents a loss of earnings to farmers, notably when cultivation space is limited. In this case, a human-driven transition between a dioecious state in wild individuals to a monoecious-like state (i.e., gynodioecious, andromonoecious, hermaphroditic) in cultivated individuals is often observed [9,10,30]. In other words, individuals showing an intermediary sexual system with a mix of male/female or hermaphroditic flowers would be favored by farmers as compared to individuals bearing only male flowers. After multiple generations of selection, pure male individuals could basically disappear from the cultivated compartment to be replaced by intermediary sexual forms. 

The genus *Dacryodes* is described as dioecious [20]. However, it is well known that cultivated *D. edulis* individuals bear either female flowers only or a mix of male and hermaphrodite flowers in various proportions [21]. The sex distribution in wild *D. edulis* individuals has never been studied before, and it is still unclear if a domestication syndrome effect can be observed on the sex distribution of cultivated individuals. Our results indicated a slight decrease of the number of purely male individuals in the cultivated compartment as compared to the wild compartment. Accordingly, we had a relative increase of purely female and hermaphrodite individuals in the cultivated compartment in agreement with expectations. This result would need to be confirmed at a larger spatial and temporal scales and by characterizing more individuals and more inflorescences per individual for many flowering cycles. It would be particularly interesting to study sex distribution along a domestication gradient to confirm our preliminary findings.

We did not observe any clear variation pattern between flowers’ morphometric parameters measured for wild and cultivated trees. The differences that were observed (stamen and peduncle length of male flowers, stamen and ovary length of female flowers) did not seem to reflect any trends between wild and cultivated individuals, but rather differences among sites without a clear relationship with the status of the tested individuals. Studies of Ferrer et al. [29] on the domestication syndrome of three native species in Mayan home gardens showed that in *Cordia dodecandra* DC., a species valued for its fruits and flowers, the flowers in home garden individuals were 1.7 times larger than those of forest individuals. The increase in size of some organs such as reproductive structures in home gardens is in agreement with expectations for trees that would have been selected for their edible fruits and can be interpreted as a domestication syndrome resulting from the history of cultivation of the species and human selection [31,32]. One of the factors responsible for an increase of organ size in the cultivated compartment can be the lower level of environmental stress [11], which, in turn, would allow a greater resource allocation to flowering/fructification. Altogether, the relationship between female flower size (i.e., ovary size) and fruit size does not seem to be straightforward, and would require further investigation, particularly at the intraspecific level.

### 3.2. Effect of Selection on the Morphometric Parameters of Fruits and Seeds and on the Germination Dynamic

We observed a clear signal of domestication syndrome at the level of fruit morphometric characteristics: fruits from wild trees have a smaller mass, length, width and thickness than those from cultivated ones. Fruits’ morphology is one of the main traits of interest to farmers and consumers. It has been demonstrated that a large variation in shape, size, color and taste of the fruits exist in cultivation, as observed in our study and as already observed in previous studies [25,27] based on a larger sample size. The results obtained here support the hypothesis that the observed variation could be by human practices, with overall a tendency to select mostly individuals with bigger fruits. Such a variation based on the size of the edible parts is common to most domesticated crops [11,13,31,32,33,34]. 

The same trend (bigger seeds in cultivated than in wild individuals) was also observed on seeds’ morphometric characteristics. This might illustrate the strong selective environmental stresses acting on wild individuals in the forest as compare to more conducive environment offered in cultivated stands [11]. The domestication syndrome observed here is probably a by-product of the selection done on fruit morphometry, rather than a direct selection for bigger seeds: by selecting for bigger fruits, farmers indirectly select for bigger seeds. Interestingly, the relationship between seed and fruit mass is stronger in wild individuals than in cultivated ones. The correlation between seed and fruit size was observed in *Chrysophyllum cainito* L. and justified by the physiological phenomena where developing seeds are a source of auxin for fruit development, so an increase in seeds will result into more auxin source and therefore larger fruit growing [13]. An increase of seed size during the domestication syndrome [35,36] was observed for many species such as *Phoebe cooperiana* P. C. Kanjilal & Das. [15], *Phoenix dactylifera* L. (date Palm) [30] or *Malus* spp. (apple) [33].

We did not observe any domestication syndrome on the germination rate and temporal dynamic in *D. edulis*. The relatively larger number of seeds from wild individuals germinating during the period 34–40 days after seed sowing might indicate an ecological difference between wild and cultivated environments, with the necessity to develop leaves faster for seedlings from wild individuals which generally grow under the canopy of other trees (forest habitat). This difference did not seem to be linked to the number of cotyledon lobes that seemed to be decamerous in most individuals, whatever their wild or cultivated status. Moreover, the large variation on the number of cotyledon lobes (from 4 to 16 cotyledon lobes) was observed both in seeds from cultivated and wild trees without any clear difference. In our study, it could be suggested that human selection did not influence the germination pattern in *D. edulis* seeds. Similar results have been found in study of Cacti species [37] in Mexico where the seed germination of wild and cultivated populations was showing similar patterns. 

### 3.3. Effect of Selection on the Lipid Composition of D. edulis Fruits

Human selection practices did not seem to have influenced the oil content and fatty acid composition of fruits from *D. edulis* cultivated individuals. The only difference in fruits composition between wild and cultivated individuals was observed for palmitic acid, which was more abundant in wild fruits than in cultivated ones. The fruits of *D. edulis* studied contained a high amount of lipids (but highly variable among individuals, 46–91%), palmitic, oleic and linoleic acids, confirming previous results obtained in different countries [38,39,40,41,42]. Although local people favored oily fruits during the cultivation of the species, this selection did not seem to have influenced the fatty acid composition of the fruits from cultivated trees. Besides the size and the lipid content of the fruits, their taste is one of the main characters under selection. The fruits of cultivated *D. edulis* presented a great variation in sourness, and consumers’ preferences is usually for non-sour fruits [25]. The same variation seemed to exist in wild fruits (data not shown). Given the absence of significant differences in the oil content/fatty acid composition of wild and cultivated fruits, our results suggested that there was no relationship between the taste of the fruits and their lipid composition. This result is in line with previous findings showing no of relationship between mesocarp test (sourness/non-sourness) and oil richness of the fruits [43]. The selection based on the taste of the fruits might have affected other biochemical characteristics of the fruits, such as hydrophilic compounds that confer sourness. A better interpretation of these results would also require getting an estimate of the proportion of sour/non-sour fruits in wild versus cultivated populations of the species. Moreover, oil extraction from *D. edulis* has not yet been reported among the species uses. Therefore, the absence of a domestication syndrome linked to oil biochemical composition of *D. edulis* mesocarp is not surprising and the same observation was found in olive trees [34,44,45]. 

## 4. Material and Methods

### 4.1. Study Sites

The study was conducted in the Centre, East and South regions of Cameroon where wild and cultivated individuals of *D. edulis* can be found (Figure 1). We classified individuals as wild when they were found in the middle of the forest, with no trace of ancient village, and no evidence of past or present species management as attested by local people. To the contrary, cultivated individuals corresponded to trees managed by local people in agroforestry systems. Hereafter, we used the wording “*status of individuals*” to refer to the wild or cultivated status of the sampled trees. Due to practical reasons, notably the availability of sufficient wild fruits to be collected, the different traits investigated were not always measured at the same sites (Figure 1). Details are provided in the following parts.

### 4.2. Sex Distribution and Flower Morphometric Characteristics

For this experiment, 49 cultivated trees were sampled in Makak (N = 16), Messock (N = 3) and Eseka (N = 30), and 38 wild trees in Messock (N = 28) and Somalomo (N = 10) (Figure 1), making a total of 87 flowering trees sampled. For each of them, the geographical coordinates and dendrometric parameters (height and diameter at breast height, dbh) were recorded. Two types of flowering material were collected according to the state of inflorescence. Whole inflorescences were collected and stored as a herbarium specimen (N = 79). However, for trees on which fruit setting was already initiated, only apical flowers were collected and conserved in 40° alcohol (N = 8). Dried flowers were softened by soaking them in lukewarm water for 10 to 15 min before being dissected, whereas flowers preserved in alcohol were observed directly. The dissected flowers’ parts were observed with a binocular magnifying glass (1× to 2× magnification) and described following Kengue [21] through the identification of the sexual type of flowers (male, female or hermaphrodite) and the estimation of their proportion per inflorescence for each sampled individual (Figure S6). Fifteen flowers were observed per inflorescence (30 flowers in total per tree). The following morphometric parameters were measured on each flower: total length of the flower (base of flower stalk to top), peduncle, stamens and ovaries [46]. Data were grouped by inflorescence, tree and status of individuals. ANOVA and Tukey tests were performed to compare the means of the flower’s morphometric parameters among inflorescences, trees and statuses of individuals. MFA was performed to understand the relationship between the qualitative and quantitative variables. The qualitative variables were the status of individuals and the sites, and the quantitative variables were the number of flowers per inflorescence (small and large), inflorescence length (small and large), ovary and stamen length (of small and large inflorescence) and the proportion of sexual type (male, female or hermaphrodite). 

### 4.3. Fruit and Seed’s Morphometric Characteristics

Fruits collection was carried out over two consecutive years (2019 and 2020). Due to the small number of wild trees bearing fruits in the four study sites, we extended our sampling zones for fruit collection to other sites. 10 to 20 mature fruits were collected per tree and stored into labelled nets allowing air to pass through to minimize rotting during their transportation. Fruits from cultivated trees were sampled in Makak (N = 263), Eseka (N = 388), Somalomo (N = 471), Messock (N = 529), Akom II (N = 239) and Campo-Ma’an (N = 26), and those from wild trees in Bipindi (N = 191), Yokadouma (N = 179), Somalomo (N = 74), Messock (N = 171), Akom II (N = 3) and Eseka (N = 14) (Figure 1). Trees were randomly selected and separated by at least three meters to avoid joined crowns for the cultivated trees [13]. The descriptors developed by Leaket et al. [47] and Anegbeh et al. [48] were used to characterize the fruits and seeds of *D. edulis* (Figure S7). For each fruit, the mass was measured, the length and width of the whole fruit were measured using a caliper, and the shape was assessed using the standardized templates described by Ndindeng et al. [49] for fruits of *D. edulis* in Cameroon. The fruits were split into two parts and the thickness of the pulp was measured using a caliper [49]. Using a knife, the seeds were carefully removed from the fruits. For the seeds, the mass, width and length were measured, and the number of cotyledon lobes were counted. ANOVA and Tukey tests were used to compare the means of the morphometric parameters according to the status of individuals and their site of provenance. A FAMD was performed to characterize the relationships between fruit (length, width, mass, pulp mass and thickness) and seed (length, width, mass and number of cotyledon-lobes) variables. 

### 4.4. Seed Germination Tests

A subpart of the seeds extracted from the fruits used for the morphometric study (Section 4.3 above) was tested for germination: fruits collected in Messock (N = 157 from 8 wild trees and 393 from 13 cultivated trees), Somalomo (N = 40 from 2 wild trees and 471 from 27 cultivated trees) and Eseka (N = 386 from 20 cultivated trees). Thus, in total 197 seeds from wild trees and 1250 seeds from cultivated trees were used for the germination tests (Table S2). Seeds from fruits collected at other sites (Bipindi, Akom II, Campo-Ma’an, Yokadouma) were characterized in situ and excluded from the germination tests as their viability could not be assured over time (more than 10 days after the collection of seeds). A germinator frame was constructed and filled with fine sand used as a germination substrate [22]. The main treatments tested were the site of origin and the status of the mother trees (wild or cultivated). The experimental design was a completely randomized block design (each block was formed by wild and cultivated seeds and sub-plots by sites). A seed was considered germinated with the appearance of the first leaves corresponding to the third stage of seed germination [21]. The germination rate was determined for wild and cultivated individuals at regular time intervals (after 28, 31, 34, 37, 40, 43 and 46 days). The difference in the dynamic of germination rate between seeds from wild and cultivated trees was tested at each of these time intervals using a *t*-test. FAMD was performed to characterize the relationships between morphometric variables of seeds, sites and germination time. 

### 4.5. Oil Content and Fatty Acid Composition of D. edulis Fruits

Fruits from 51 trees (35 cultivated + 16 wild) with ten to twenty fruits per tree were used for the lipid analyses (Figure 1). After a morphological characterization of the fruits (Table S3), the mesocarp (pulp) was separated from the seed by vertical sectioning of the fruit with a knife. The interior of the pulp was cleaned, dried for 48 h at 45 °C, and a portion of each dried fruit was ground to powder. The oil content of the mesocarp (% of dry matter) was determined according to the International Union of Pure and Applied Chemistry (IUPAC) method [50]. Total lipids were extracted with hexane by hot reflux in a Soxhlet apparatus using 15 g of fruit powder. Oil samples were stored at –20 °C until the FAs was analyzed.

Fatty acid methyl esters (FAME) were prepared according to the ISO-5509 standard from 30 mg oil samples and analyzed using gas chromatography to determine the FA composition [51]. Two FAME analyses were carried out per oil sample. Analyses were performed using an Agilent 7820A gas chromatography system with flame ionization detection. A Restek Famewax capillary column (30 m × 0.25 mm × 0.25 m) was used. The carrier gas was helium at 40 cm/s. Analyses were conducted from 185 °C to 225 °C at 4 °C/min and then kept at 225 °C for 10 min. Only peaks identified in the chromatograms with a relative percentage >0.1% were retained. ANOVA and Tukey test were used to compare the mesocarp oil content and FA composition of wild and cultivated individuals. PCA was performed to discriminate individuals along quantitative variables. 

All data were analyzed using R.4.0.4 software [52], using R packages such as FactoMineR, Factoextra, data.table, missMDA, Factoshiny, calibrate and agricolae. A two-way ANOVA was used to compare means between groups and the Tukey test for their separation; differences were considered significant at the 5% probability level. 

## 5. Conclusions

*Dacryodes edulis* presents a set of characteristics that constrain the domestication of the species: a long generation time, predominantly allogamous species and propagation by seeds. These characteristics represent strong impediments to farmers to fix their characters of interest. However, we observed differences on two set of traits important for farmers: the sex distribution—with a higher representation of the male function in wild populations than in cultivated ones—and on the morphometric characteristics of the fruits and seeds—with a smaller mass/size in wild individuals. These two traits are important for farmers given their links to productivity (higher number of productive trees) and economic values (fruits corresponding to consumers demand). For an extensive study of the domestication syndrome in the species, it will be particularly interesting to investigate the genetic differences between wild and cultivated *D. edulis* populations.

## Figures and Tables

**Table 1:** Distribution of sexes among cultivated and wild individuals.

	Cultivated Individuals			Wild Individuals		
Sexual Type	Eseka (N = 30)	Makak (N = 16)	Total (N = 46)	Messock (N = 28)	Somalomo (N = 10)	Total (N = 38)
Male	5 (16.7%)	3 (18.7%)	**8 (17.4%)**	11 (39.3%)	7 (70.0%)	**18 (47.4%)**
Female	12 (40.0%)	4 (25.0%)	**16 (34.8%)**	7 (25.0%)	1 (10.0%)	**8 (21%)**
Male-hermaphrodite	5 (16.7%)	7 (43.8%)	**12 (26.1%)**	10 (35.7%)	2 (20.0%)	**12 (31.6%)**
Hermaphrodite	8 (26.6%)	2 (12.5%)	**10 (21.7%)**	0	0	**0**

**Table 2:** Comparison of flower morphometric parameters in *D. edulis* according to the sexual type, status of individuals and sites.

Factors	Modalities	N	Flower Length (mm)	Stamen Length (mm)	Peduncle Length (mm)	Ovary Length (mm)
	**Male flowers**					
Status of individuals	Cultivated	240	5.36 ± 1.34 ^a^	2.05 ± 0.59 ^a^	1.41 ± 0.68 ^a^	0.05 ± 0.19 ^a^
	Wild	540	5.52 ± 1.42 ^a^	1.90 ± 0.58 ^b^	1.68 ± 0.78 ^b^	0.05 ± 0.18 ^a^
Sites	Eseka (C)	150	5.27 ± 1.12 ^a^	2.06 ± 0.57 ^a^	1.25 ± 0.51 ^c^	0.07 ± 0.24 ^a^
	Makak (C)	90	5.51 ± 1.63 ^a^	2.03 ± 0.62 ^a^	1.68 ± 0.83 ^ab^	0.00 ± 0.0 ^b^
	Messock (W)	330	5.21 ± 1.14 ^a^	2.03 ± 0.51 ^a^	1.59 ± 0.71 ^b^	0.08 ± 0.22 ^a^
	Somalomo (W)	210	6.01 ± 1.66 ^b^	1.69 ± 0.62 ^b^	1.82 ± 0.87 ^a^	0.00 ± 0.00 ^b^
	**Female flowers**					
Status of individuals	Cultivated	530	7.40 ± 1.42 ^a^	1.47 ± 0.77 ^a^	2.86 ± 1.02 ^a^	2.36 ± 0.70 ^a^
	Wild	270	7.43 ± 1.42 ^a^	1.36 ± 0.5 ^b^	2.81 ± 0.96 ^a^	2.23 ± 0.63 ^b^
Sites	Eseka (C)	350	7.54 ± 1.51 ^a^	1.53 ± 0.86 ^a^	3.01 ± 1.01 ^a^	2.46 ± 0.63 ^a^
	Makak (C)	120	7.25 ± 1.83 ^ab^	1.23 ± 0.58 ^b^	2.78 ± 1.13 ^a^	2.11 ± 0.91 ^b^
	Messock (C)	60	6.81 ± 1.04 ^b^	1.57 ± 0.35 ^a^	2.23 ± 0.54 ^b^	2.31 ± 0.40 ^ab^
	Messock (W)	240	7.47 ± 1.75 ^a^	1.43 ± 0.5 ^ab^	2.85 ± 0.96 ^a^	2.34 ± 0.56 ^a^
	Somalomo (W)	30	7.13 ± 1.22 ^ab^	0.85 ± 0.45 ^c^	2.50 ± 0.90 ^ab^	1.38 ± 0.44 ^c^

Mean ± standard deviation (based on measurements of flower) followed by the letter are significantly different at the 5% probability level; (C) indicates cultivated individuals; (W) wild individuals.

**Table 3:** Fruits and seeds morphometric characteristics in wild and cultivated populations of *D. edulis*.

	Status of Individuals		Morphometric Parameters				
**Fruits**		N	Mass (g)	Length (mm)	Width (mm)	Thickness (mm)	Mass of pulp (g)
	Cultivated	1896	49.10 ± 17.52 ^a^	68.24 ± 14.0 ^a^	36.20 ± 7.74 ^a^	5.70 ± 1.42 ^a^	36.60 ± 15.70 ^a^
	Wild	650	15.60 ± 7.30 ^b^	42.04 ± 8.41 ^b^	23.00 ± 4.41 ^b^	2.72 ± 1.30 ^b^	9.72 ± 5.01^b^
**Seeds**		N	Mass (g)	Length (mm)	Width (mm)	N cotyledon lobes	
	Cultivated	1896	12.32 ± 4.07 ^a^	40.60 ± 12.80 ^a^	21.56 ± 5.95 ^a^	9.6 ± 1.04 ^a^	
	Wild	650	6.05 ± 2.63 ^b^	32.30 ± 5.83 ^b^	15.40 ± 3.45 ^b^	9.34 ± 1.46 ^b^	

Mean and ± standard deviations measurements of fruits and seeds. For the same columns, different letters represent a significant difference at the 5% risk level.

**Table 4:** Fruit mesocarp total oil content and fatty acid composition in cultivated and wild *D. edulis*.

	Status of Individuals		
Compounds	Cultivated	Wild	F Value
%Oil	67.10 ± 11.80	64.30 ± 11.30	0.8
%Palmitic acid	39.62 ± 4.05	41.96 ± 2.64	6.5 *
%Palmitoleic acid	0.17 ± 0.07	0.15 ± 0.05	0.9
%Stearic acid	2.64 ± 0.75	2.46 ± 0.75	0.6
%Oleic acid	32.52 ± 6.15	32.62 ± 6.04	0.01
%Vaccenic acid	0.95 ± 0.25	0.98 ± 0.34	0.2
%Linoleic acid	23.11 ± 20.76	20.76 ± 5.42	3.1
%Linolenic acid	0.82 ± 0.25	0.91 ± 0.17	1.7

* Significantly different at *p* = 0.05 (Tukey test).

## Data Availability

Data that support this study are available from the corresponding author upon request.

## Supplementary Materials

The following supporting information can be downloaded at: https://www.mdpi.com/article/10.3390/plants11192496/s1, Figure S1: (A) female flowers, (B) hermaphrodite flowers, (C) male flowers, (D) male flowers with reduced ovary, (E) morphology of a wildflower, (F) morphology of a cultivated flower, (G) morphology of the ovary and stamin of a cultivated female flower, (H) morphology of the ovary and stamin of a wild flower (magnification: 2×), Figure S2: (A) Discrimination of wild and cultivated individuals along the two axes according to gender, population. (B) Correlation of variables to the formation of axes 1 and 2. (C) Separation of individuals along axis 1 according to sites. (D) Separation of populations according to the height of individuals, Figure S3: Distribution of the number of cotyledon lobes between wild and cultivated seeds, Figure S4: (A) Discrimination of wild and cultivated fruits from the FAMD according to axes 1 and 2. (B) Correlation and contribution of variables to the formation of axes 1 and 2, Figure S5: (A) Segregation of individuals along the two axes according to individual status and sites. (B) Correlation of variables to the formation of axes 1 and 2. Figure S6: Descriptors developed by Kengue (1990) for the flowers, Figure S7: Descriptors of fruits and seeds morphometric characteristics of *D. edulis* (Ndindeng et al., 2008), Table S1: Morphometric characteristics of fruits and seeds between sites, Table S2: Seed characteristics used for the germination test, Table S3: Morphometric characteristics of wild and cultivated fruits’ pulp of safou.
