# Supplementary material for: Domestication Syndrome in Dacryodes edulis (Burseraceae): Comparison of Morphological and Biochemical Traits between Wild and Cultivated Populations
Source: Plants (Basel). 2022 Sep 23;11(19):2496. doi: 10.3390/plants11192496 (PMC9571564; doi:10.3390/plants11192496)
Supplement: Supplementary file 1 [file plants-11-02496-s001.zip › plants-1865551-supplementary.pdf]

SUPPLEMENTARY DATA

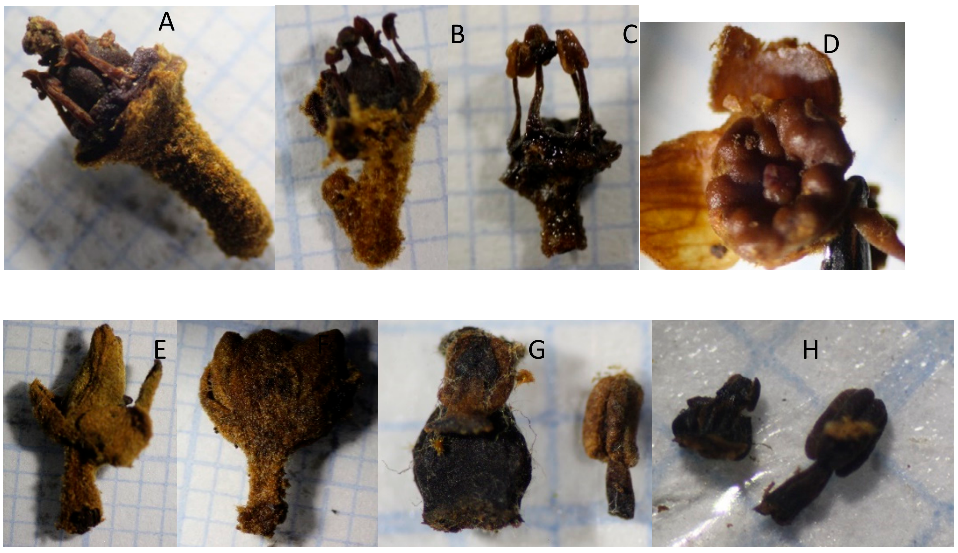

Figure S1. (A) female flowers, (B) hermaphrodite flowers, (C) male flowers, (D) male flowers with reduced ovary, (E) morphology of a wild flower, (F) morphology of a cultivated flower, (G) morphology of the ovary and stamin of a cultivated female flower, (H) morphology of the ovary and stamin of a wild flower (magnification: 2X).

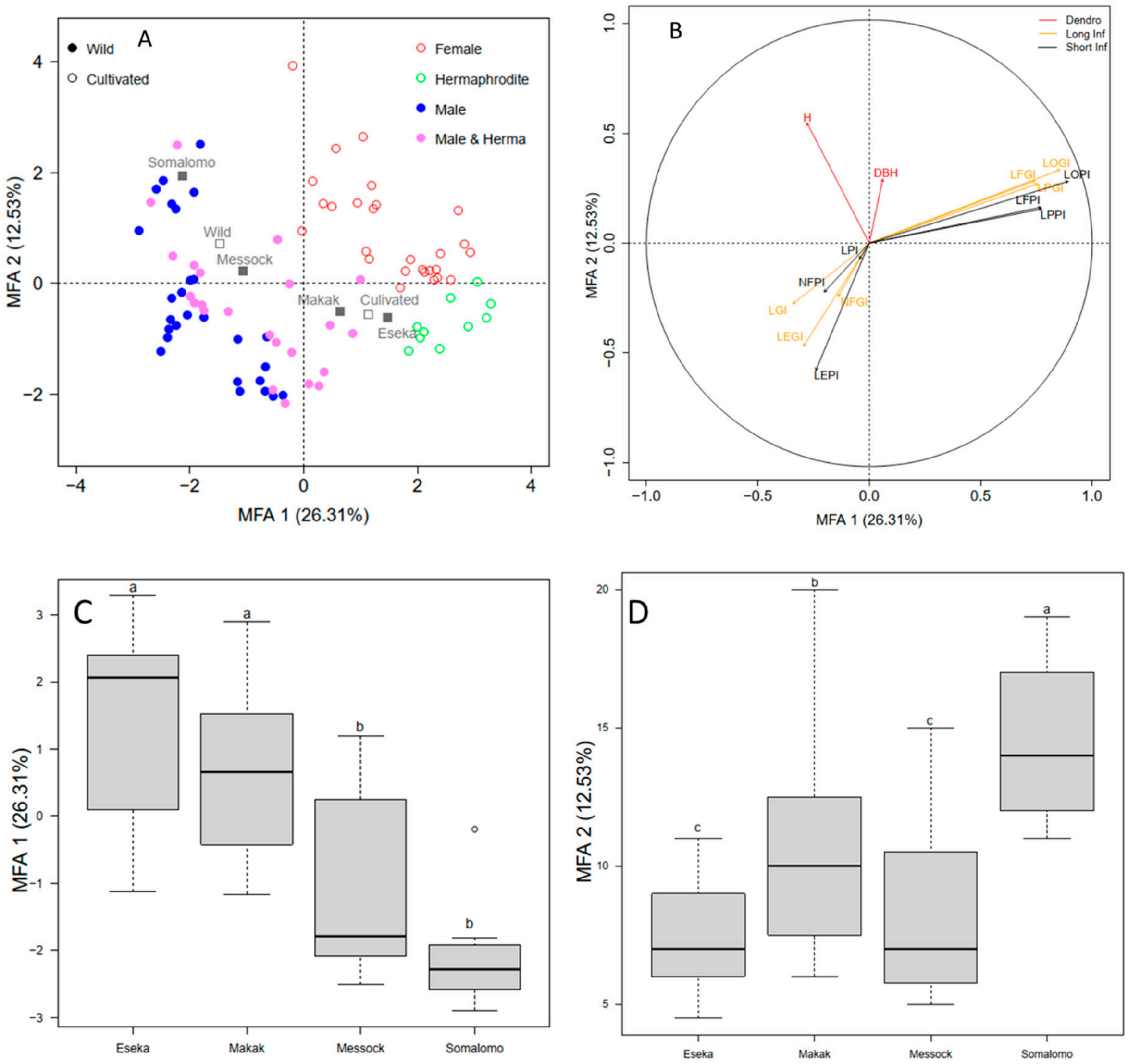

Figure S2. A. Discrimination of wild and cultivated individuals along the two axes according to gender, population. B. Correlation of variables to the formation of axes 1 and 2. C. separation of individuals along axis 1 according to sites. D. separation of populations according the height of individuals. (LPPI: peduncle length small inflorescence, LOPI: ovary length of small inflorescence, LEPI: stamin

length of small inflorescence, LFPI: flower length of small inflorescence, NFPI: number of flowers of small inflorescence, LPI: length of small inflorescence, LOGI: ovary length of large inflorescence, LPGI: peduncle length of large inflorescence, LEGI: stamin length of large inflorescence, LFGI: flower length of large inflorescence, NFGI: number of flowers of large inflorescence, LGI: length of large inflorescence, Hau: height, DBH: diameter)

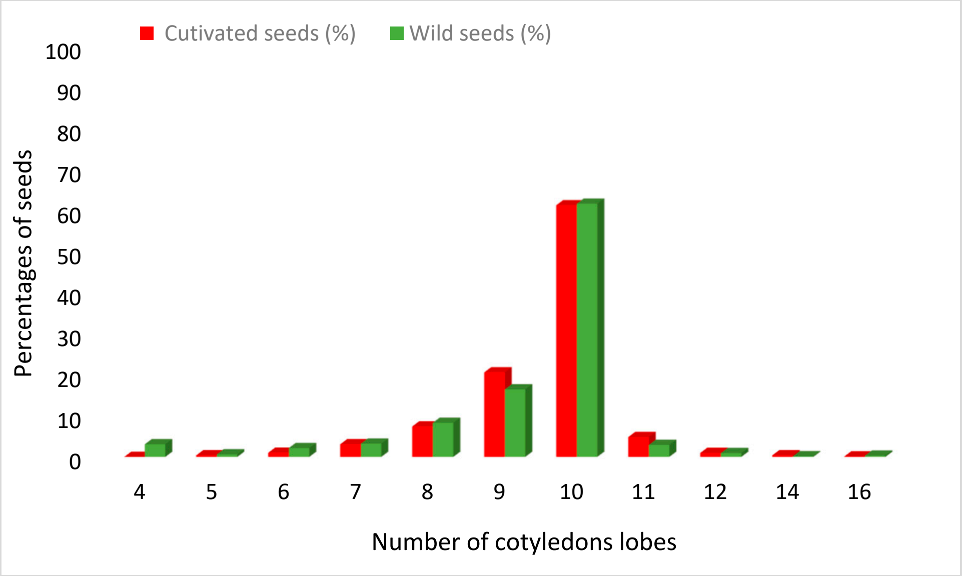

Figure S3. Distribution of the number of cotyledon lobes between wild and cultivated seeds

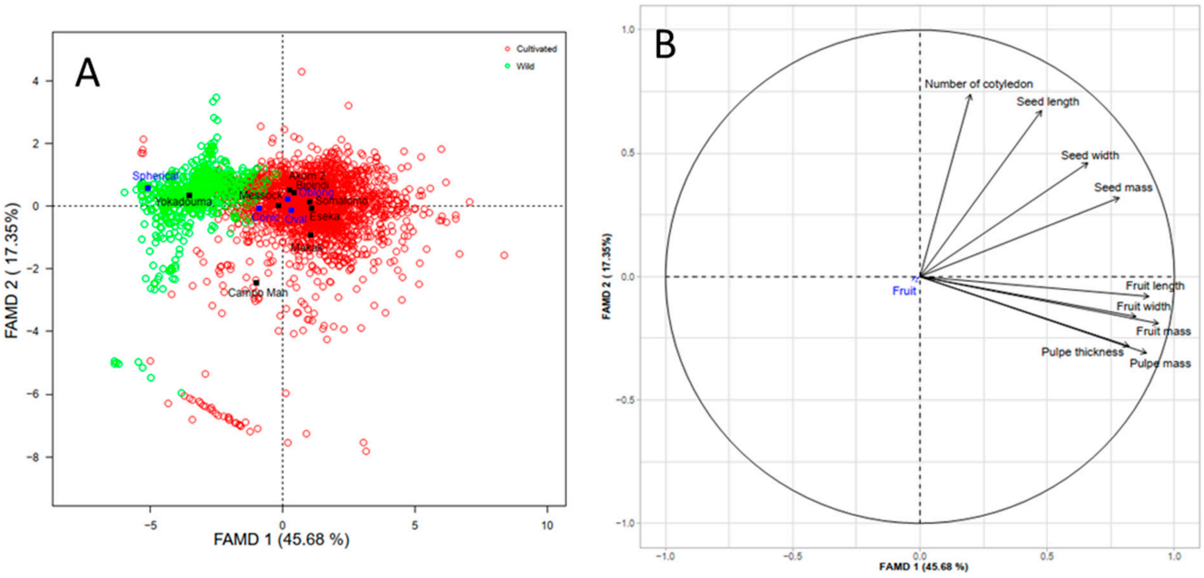

Figure S4. A. Discrimination of wild and cultivated fruits from the FAMD according to axis 1 and 2. B. Correlation and contribution of variables to the formation of axes 1 and 2.

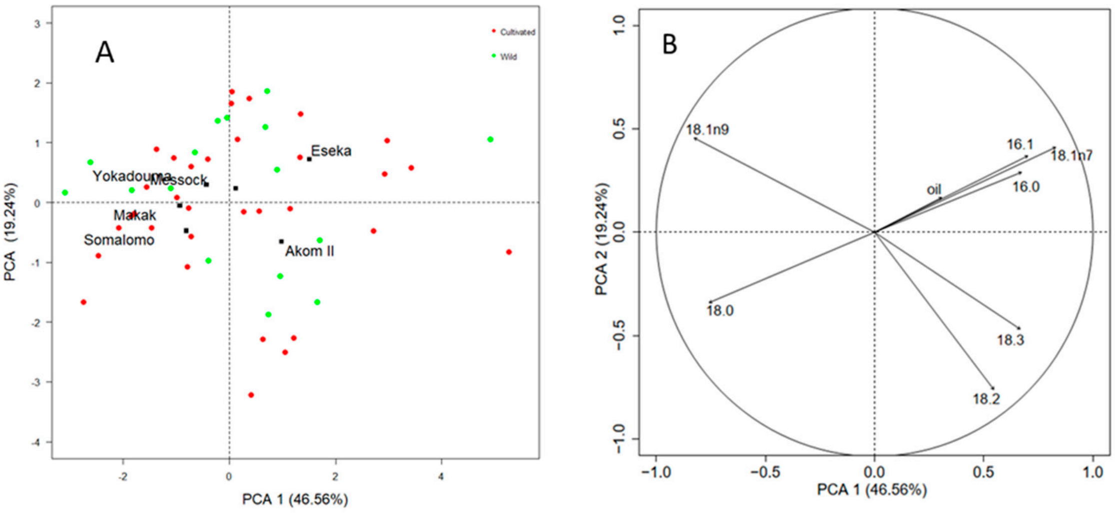

Figure S5. A. Segregation of individuals along the two axis according to individual status and sites. B. Correlation of variables to the formation of axes 1 and 2

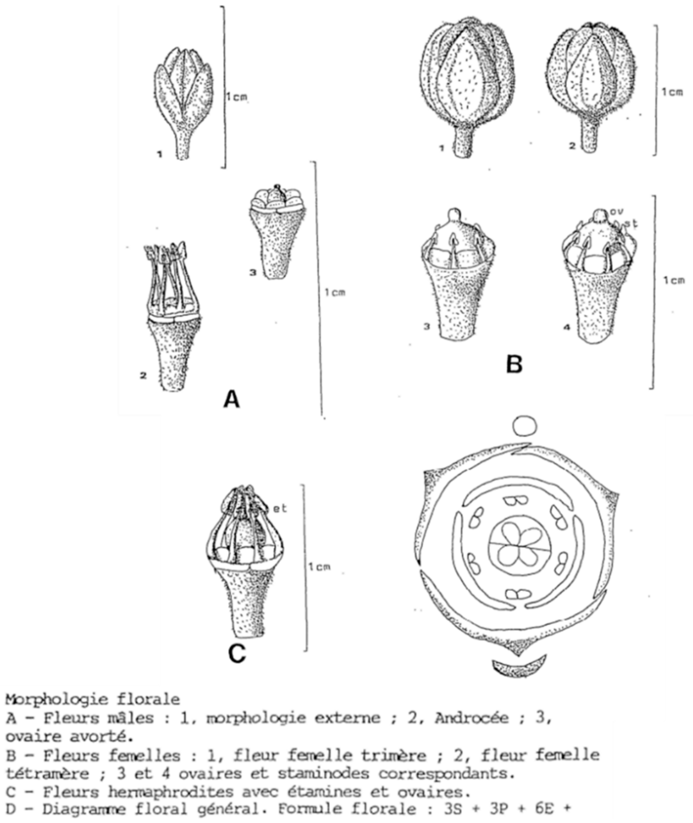

Figure S6. Descriptors developed by Kengue (1990) for the flowers

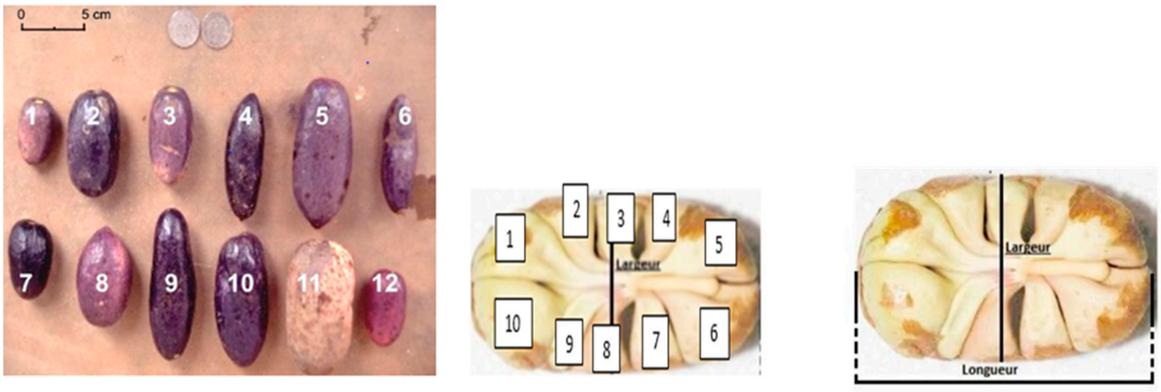

Figure S7. Descriptors of fruits and seeds morphometric characteristics of *D. edulis* (Ndindeng *et al.*, 2008)

Table S1. Morphometric characteristics of fruits and seeds between sites

sites		Morphometric parameters of fruits				
	N	Mass (g)	Length (mm)	Width (mm)	Thickness (mm)	Mass of pulp (g)
MAK	263	$56.5±16.4^{a}$	$66.5±20.2^{b}$	$35.1±10.4^{c}$	$6.3±1.3^{a}$	$44.1±15.0^{a}$
SOM	471	$52.7±19.0^{b}$	$72.6±12.6^{a}$	$38.6±9.0^{a}$	$6.1±1.3^{a}$	$39±16.4^{b}$
ESE	388	$48.1±18.4^{c}$	$68.3±11.5^{b}$	$37±6.4^{b}$	$5.7±1.2^{b}$	$36.7±16.9^{bc}$
MES	529	$45.2±14.4^{cd}$	$67.6±12.1^{b}$	$36±2.6^{bc}$	$5.8±1.2^{b}$	$34.0±13.3^{c}$
AKO	239	$42.6±16.1^{d}$	$62.4±13.2^{c}$	$32.1±5.1^{d}$	$4.1±1.4^{c}$	$29.2±13.2^{d}$
CPM	26	$39.1±8.2^{d}$	$63.0±5.0^{bc}$	$32.0±6.3^{d}$	$3.5±0.8^{cd}$	$29.5±9.3^{cd}$
ESE^{w}	14	$20.1±5.4^{e}$	$49.2±9.8^{d}$	$27.1±3.4^{de}$	$3.8±0.5^{cd}$	$13±4.8^{e}$
BIP	191	$18.1±8.0^{e}$	$44.3±9.4^{de}$	$24.6±4.7^{ef}$	$3.2±1.0^{d}$	$11.0±5.5^{e}$
YOKA	179	$15.6±5.1^{e}$	$41.4±7.5^{def}$	$21.6±4.0^{eg}$	$1.6±0.6^{e}$	$9.5±5.2^{e}$
SOM^{w}	74	$15.1±7.1^{e}$	$42.0±8.0^{def}$	$22.1±5.0^{efg}$	$3.4±2.5^{d}$	$9.3±5.2^{e}$
MES^{w}	171	$11.7±2.5^{e}$	$38.3±5.2^{df}$	$22.0±2.6^{eg}$	$3.0±2.5^{d}$	$7.0±2.0^{e}$
AKO^{w}	3	$3±0.0^{e}$	$22.7±2.5^{ef}$	$12.3±2.5^{fg}$	$1.0±0.0^{e}$	$1±0.0^{e}$
Morphometric parameters of seeds						
Sites	N	Mass (g)	Length (mm)	Width (mm)	N cotyle	
MAK	263	$12.5±3.8^{b}$	$37.3±12.0^{cd}$	$16.7±6.0^{c}$	$9.2±1.1^{ab}$	

SOM	471	13.7±4.2[a]	43.4±21.0[a]	21.6±6.1[b]	9.7±0.9[ab]
ESE	388	11.5±3.4[c]	39.6±5.5[bc]	24.8±5.6[a]	9.4±1.3[ab]
MES	529	11.3±2.5[c]	40.4±8.7[b]	21.4±5.01[b]	9.7±0.9[ab]
AKO	239	13.4±4.1[ab]	41.4±6.2[ab]	20.3±4.0[b]	9.8±0.9[ab]
CPM	26	9.4±3.6[cd]	36.4±9.6[cde]	15.9±2.5[c]	9.3±0.8[ab]
ESE[w]	14	6.8±1.3[de]	33.6±3.2[de]	17.6±2.5[bc]	10.2±0.7[a]
BIP	191	6.7±2.9[e]	34.0±5.6[de]	15.8±2.5[c]	9.6±0.6[ab]
YOKA	179	6.1±2.1[e]	30.1±4.2[e]	13.7±3.0[c]	9.2±1.3[ab]
SOM[w]	74	5.6±1.8[e]	33.3±4.5[de]	14.8±2.7[c]	9.7±0.7[ab]
MES[w]	171	4.6±1.42[e]	31.3±6.8[e]	16.5±3.4[c]	9.0±0.7[b]
AKO[w]	3	2±0.0[e]	20.3±0.6[e]	11.0±1.0[c]	10±0.0[ab]

Mean±standard deviations (measurements of fruits and seed) followed by the letter are significantly different at the 5% probability level (MAK: Makak, SOM: Somalomo, ESE: Eseka, Mes: Messock, AKO: Akom II, CPM: Campo-Ma'an, ESEs: Eseka wild fruits, BIP: Bipindi, YOKA: Yokadouma, SOM[w]: Somalomo wild fruits, MES[w]: Messock wild fruits, AKO[w]: Akom II wild fruits)

Table S2. Morphometric characteristic of wild and cultivated fruit' pulp of safou

Status of individuals	Mass (g)	Length (mm)	Width (mm)	Thickness (mm)	Mass of pulp (g)	N
Cultivated	50.08±19.65[a]	68.72±12.67[a]	35.64±6.65[a]	5.91±2.81[a]	37.78±18.97[a]	35
Wild	15.28±7.13[b]	40.69±7.88[b]	22.51±4.05[b]	2.70±1.3[b]	9.50±4.90[b]	16

Mean±standard deviations (measurements of fruits and seed) followed by the letter are significantly different at the 5% probability level

Table S3. Seed characteristics used for the germination test

Status of individuals	Mass (g)	Length (mm)	Width (mm)	N cotyledon lobes	N seeds
Cultivated	12.49±4.10[a]	41.72±14.32[a]	23.08±5.54[a]	9.61±1.18[a]	1250
Wild	5.13±1.62[b]	31.83±6.82[b]	16.60±3.33[b]	8.97±2.24[a]	197

Mean±standard deviations (measurements of fruits and seed) followed by the letter are significantly different at the 5% probability level
